# Influence of the interaction between parental myopia and poor eye habits when reading and writing and poor reading posture on prevalence of myopia in school students in Urumqi, China

**DOI:** 10.1186/s12886-021-02058-3

**Published:** 2021-08-14

**Authors:** Haonan Shi, Jing Fu, Xiaojing Liu, Yingxia Wang, Xianting Yong, Lan Jiang, Shaowei Ma, Zhe Yin, Jian Yao, Xuan Yao, Xueyi Chen, Tingting Wang

**Affiliations:** 1 School of Public Health, Xinjiang Medical University, 830011 Urumqi, China; 2 Department of Ophthalmology, First Affiliated Hospital, Xinjiang Medical University, 830000 Urumqi, China; 3 Health Care Guidance Centre in Primary and Secondary Schools, 830002 Urumqi, China; 4 School of Nursing & Health Management, Shanghai University of Medicine & Health Sciences, 201318 Shanghai, China; 5 Maternal and Child Health Care Hospital of Uygur Autonomous Region, 830002 Urumqi, China; 6 College of Medicine, Shanghai University, 200444 Shanghai, China; 7 Department of Ophthalmology, First Affiliated Hospital, Xinjiang Medical University, 830001 Urumqi, China

**Keywords:** Myopia, Heritability, Poor reading and writing habits, Interaction

## Abstract

**Background:**

To evaluate the prevalence of myopia in school students in Urumqi, China, and explore the influence of the interaction between parental myopia and poor reading and writing habits on myopia to identify the at-risk population and provide evidence to help school students avoid developing myopia.

**Methods:**

A cross-sectional survey was conducted with 6,883 school students aged 7–20 years in Urumqi in December 2019. The Standard Eye Chart and mydriatic optometry were used to determine whether students had myopia. Falconer’s method was used to calculate the heritability of parental myopia. Multivariate unconditional logistic regression models were used to analyze the risk factors for myopia and the additive and multiplicative interaction of parental myopia and poor reading and writing habits.

**Results:**

After standardizing the age of the 6,883 students, the overall prevalence rate of myopia was 47.50 %. The heritability of parental myopia was 66.57 % for boys, 67.82 % for girls, 65.02 % for the Han group, and 52.71 % for other ethnicities. There were additive interactions between parental myopia and poor reading and writing habits; among them, parental myopia and poor eye habits when reading and writing (the distance between the eyes and book is less than 30 cm when reading and writing, fingers block the sight of one eye while holding the pen, and leaning one’s body when reading and writing; habit 1) increased the risk of myopia by 10.99 times (odds ratio [*OR*] = 10.99, 95 % confidence interval [*CI*] = 8.33–14.68), parental myopia and poor reading posture (reading while lying down, walking, or in the car; habit 2) increased the risk of myopia by 5.92 times (*OR* = 5.92, 95 % *CI* = 4.84–7.27). There was no multiplicative interaction between parental myopia and habit 1 or habit 2 (*OR* = 0.69, 95 % *CI* = 0.44–1.08; *OR* = 0.89, 95 % *CI* = 0.66–1.21, respectively).

**Conclusion:**

The prevalence of myopia among students in Urumqi, Xinjiang is relatively high. The risk of developing myopia is affected by parental myopia and poor reading and writing habits. In addition, parental myopia amplifies the harm caused by poor reading and writing habits, thereby increasing the risk of myopia. Students with parents who have myopia should be targeted during myopia prevention efforts.

**Supplementary Information:**

The online version contains supplementary material available at 10.1186/s12886-021-02058-3.

## Background

Myopia is a type of refractive error characterized by worsened distance vision caused by elongation of the axis oculi. With the increase in study-induced stress and the popularity of mobile electronic equipment, the prevalence of myopia has increased among school students; this phenomenon is particularly obvious in Southeast Asian countries such as China, Japan, Singapore, and Korea [1]. A survey on health and nutrition from Korea reported that 65.4 % of the population aged 5–18 years in Korea had myopia, and 8.0 % of them had severe myopia in 2016–2017 [2]. A survey of 1416 school students from Japan showed that the prevalence rates of myopia in elementary school and junior high school students were 76.5 and 94.9 %, respectively [3]. The prevalence of myopia in the Chinese was also high; a survey of the prevalence of myopia among school students in six cities showed that the overall prevalence of myopia was 55.7 % [4]. Although the prevalence of myopia is lower in the Chinese than in Korean and Japanese populations, it is still not possible to ignore the detrimental effects of myopia in the Chinese, especially in school students. In addition, we checked the results of the physique and health survey of Chinese students from 2000 to 2014; it is evident that the prevalence of poor vision is increasing in Chinese students, with myopia as the most common issue [5–8], which gradually affects younger students [9]. Therefore, the new cases of myopia demonstrate that prevention of myopia has worsened.

Genetic and environmental factors are major factors in myopia and have been accepted by most scholars [10, 11]. Recently, many studies have verified these views [12, 13]. A survey of 7,681 primary and secondary school students in Yunnan, China, a province with a multi-ethnic population, showed that the prevalence of myopia in Dai groups was higher than that in other ethnic groups (Han, Bai, Yi, and other ethnicities) [14]. Similar to Yunnan, Xinjiang is a region where multiple ethnic groups, e.g., Han, Uygur, Kazakh, and Hui, live with great differences in their lifestyles, which has caused large differences in the prevalence of myopia in different ethnic groups. However, the results opposed those obtained in Yunnan; the difference in Xinjiang was mainly manifested between the Han population and other ethnic groups. Our research team previously carried out a survey of myopia in school students in 2012 and 2016. The survey involved two cities in Xinjiang, Urumqi and Yining. The results of this research show that the prevalence of myopia in the Han population was higher than that in other ethnic groups [15–17]. To understand the change in the myopia rate in students and the influence of parents’ myopia and poor reading and writing habits, we conducted a survey again in Urumqi of Xinjiang in December 2019. Through this survey, we hoped to be able to explore the population susceptible to myopia and advise schools and parents to prevent students’ myopia.

## Methods

### Study population

This study was a school-based eye survey conducted in Urumqi, the capital city of Xinjiang, in December 2019. There are seven districts and one county in Urumqi. According to the statistics at the end of 2018, Urumqi had a population of approximately 3.5 million, which included Han, Uighur, Kazakh, Hui, and other ethnicities.

At first, we contacted the Primary and Secondary School Health Care Guidance Center and Education Bureau in Urumqi, obtained their approval, and asked school doctors in each school for their assistance. Next, we used stratified cluster sampling to randomly select four districts (Xinshi, Tianshan, Shayibaket, and Shuimogou) in Urumqi. We randomly selected students from the second grade of primary school to the second grade of high school from two to three schools in each district; a total of 7,587 students were initially included in our study. After excluding those who did not meet the inclusion criteria and who had incomplete data, 6,883 (90.72 %) school students participated in our study.

Our research adhered to the tenets of the Declaration of Helsinki for research involving human subjects and was approved by the Institutional Review Board of the First Affiliated Hospital of Xinjiang Medical University. The research methods were carried out in accordance with the approved guidelines. Written informed consent was obtained from all participating students, parents, and the head teacher, and we promised to keep their information confidential.

### Clinical eye examination

The ophthalmic examinations were conducted in each school by school doctors and an Optometrist from the First Affiliated Hospital of Xinjiang Medical University. First, school doctors used a new national standard visual acuity chart to examine uncorrected eyesight in students. Students who had uncorrected eyesight ≥ 5.0 according to the International Standard Eyesight Chart were classed as emmetropic; students with uncorrected eyesight < 5.0 underwent optometry (streak retinoscope YZ24 [Suzhou Liuliu Vision Technology Co., Ltd.{http://www.66vision.com}]) after mydriatic eye drops were administered (tropicamide, purchased from Tianjin Jinyao Group Hebei Yongguang Pharmaceutical Co. Ltd. [http://www.ieye.com.cn]). Students with SE <-0.50 diopters were diagnosed with myopia [18]. If there was a difference between the two eyes, the eye with poorer vision was chosen as the standard. Students with hyperopia, ocular trauma, trachoma, anisometropia, corneal disease, glaucoma, cataract, uveitis, retinal diseases, or other ocular diseases were excluded. In addition, students with diabetes, hyperthyroidism, and endocrine-related metabolic diseases were also excluded.

### Measurement of covariates

We designed a questionnaire to examine the characteristics of students in Xinjiang with the help of head teachers and school students. All students who agreed to participate in the survey answered the questionnaire in a classroom at a class meeting. The questionnaire included the following: (1) general demographic characteristics (sex, ethnicity, age, grade, family history of myopia, parental education, family income, place of residence [urban or rural]); (2) vision-related conditions (age when first developed myopia, frequency of changing glasses, history of previous eye diseases, etc.); and (3) eye use (reading and writing habits; frequency of using electronic products such as computers, smart phones, tablets; sleeping time; outdoor activity time; etc.). Information on family income, parental myopia status, and ocular disease history received prior to enrollment was obtained directly by using the student national study number. During the investigation, we arranged at least 2 subject group trained investigators to cooperate with the investigation for each investigated class, which ensured the accuracy of the findings.

### Judgment criteria for other indicators

Poor eye habits when reading and writing (habit 1) was defined as maintaining a distance of < 30 cm between the eyes and book while reading and/or writing, covering the sight of one eye with the finger holding a pen while writing, and/or tilting the body when reading and/or writing.

Poor reading posture (habit 2) was defined as reading while lying down, walking, or in a moving car.

### Statistical analysis

The data were input using Epi Data 3.1 software. The chi-square test was used to examine differences in the prevalence of myopia stratified by sex, ethnicity, age, parental myopia, and poor reading and writing habits; multivariate logistic regression was used to analyze the associations of parental myopia and poor reading and writing habits with myopia, controlling for age, sex, ethnicity, and other confounding factors. The multiplicative and additive interactions between them were also analyzed. In all statistical analyses, two-tailed tests and a 5 % significance level were applied. The analyses were performed using R Studio.

Parental heritability was examined using Falconer’s formula (*h*^2^ = *b*/*r*); in this formula, *r* is the correlation coefficient (parents are first-degree relatives, *r* = 0.5), *b* is the regression coefficient, *b* = *p*_c_(*X*_c_-*X*_r_)/*a*_r_, *p*_c_=1-*q*_c_, where *q*_c_ is the prevalence of myopia in relatives in the control group, *X*_c_ is the standard deviation between the average susceptibility of relatives in the control group and the threshold, *X*_r_, is the standard deviation between the average susceptibility of relatives in the proband group and the threshold, *a*_r_, is the standard deviation between the average susceptibility of patients and their relatives in the proband. The values of *X*_c_, *X*_r_, and *a*_r_ can be found in the Falconer table.

## Results

A total of 6,883 students comprising 3,404 (50.5 %) boys and 3,479 (49.5 %) girls aged 7–20 years (mean ± standard deviation: 12.42 ± 2.46 years) completed the ophthalmic examinations and were subsequently included in this analysis. The study was composed of a multi-ethnic population including Han (3,564, 51.8 %) and other ethnicities (3,319, 48.2 %). Considering all study participants after age standardization, 47.50 % were affected by myopia. The prevalence of myopia was higher in girls than in boys (43.5 % vs. 33.6 %) and higher in the Han population than in the other ethnic groups (53.0 % vs. 23.2 %). Myopia prevalence increased significantly with increasing age and learning level (*P* for trend < 0.01). The prevalence was the lowest in students aged 7–12 years (19.9 %) and in primary school students (19.9 %) and the highest among those aged 15–20 years (71.0 %) and in senior high school students (72.0 %). More students with myopia had habit 1 and habit 2 (42.3 and 42.8 %, respectively). The prevalence of myopia in students was higher in those with both parents with myopia than in those with only one parent with myopia and those with no parents with myopia (74.3 % vs. 55.2 % vs. 29.6 %, respectively) (Table 1).

**Table 1:** Prevalence of myopia in students by sex, ethnicity, age, learning level, parental myopia, and poor reading and writing habits

Parameters	Number	Myopia	Prevalence(%)	*χ*^2^	*P*
Sex					
Male	3404	1145	33.6	70.86	< 0.01
Female	3479	1514	43.5		
Ethnicity					
Han	3564	1889	53.0	643.85	< 0.01
Other nationalities	3319	770	23.2		
Age (years)					
7–11	3508	698	19.9	1179.06	< 0.01
12–14	2243	1157	51.6		
15–20	1132	804	71.0		
Learning level					
Primary school	3093	530	19.9	1307.97	< 0.01
Junior high school	2475	1182	44.5		
Senior high school	1315	947	72.0		
Habit 1					
Yes	5965	2522	42.3	251.13	< 0.01
No	918	137	14.9		
Habit 2					
Yes	5181	2271	42.8	239.14	< 0.01
No	1702	388	22.8		
Parental myopia					
Neither	4739	1402	29.6	573.69	< 0.01
Either	1762	973	55.2		
Both	382	284	74.3		

Habit 1: Poor eye habits when reading and writing; Habit 2: Poor reading posture

Among the 13,766 parents, 18.3 % had myopia. The heritability of parental myopia for boys was 66.57 % and that for girls was 67.82 %; the heritability of parental myopia for the Han group was 65.02 % and that for other ethnic groups was 52.71 % (Table 2).

**Table 2:** Analysis of heritability of myopia using Falconer’s method

		Number of parents	Number with myopia	Prevalence of myopia(%)	*χ*^*2*^	*P*	*q*	*X*	*a*	*h*^*2*^*(%)*
Sex										
Boys	Emmetropia^1)^	4518	532	11.78	7.04	<0.01	0.8822	1.185	1.675	
	Myopia	2290	657	28.69			0.7131	0.553	1.180	66.57
Girls	Emmetropia^1)^	3930	453	11.53			0.8847	1.200	1.688	
	Myopia	3028	884	29.19			0.7081	0.553	1.180	67.82
Ethnicity										
Han	Emmetropia^a^	3350	479	14.30	274.60	<0.01	0.8570	1.067	1.579	
	Myopia	3778	1205	31.90			0.6810	0.468	1.118	65.02
Other	Emmetropia^a^	5098	506	9.93			0.9007	1.287	1.760	
	Myopia	1540	336	21.82			0.7818	0.772	1.346	52.71

^a^Control group

**Fig. 1:**
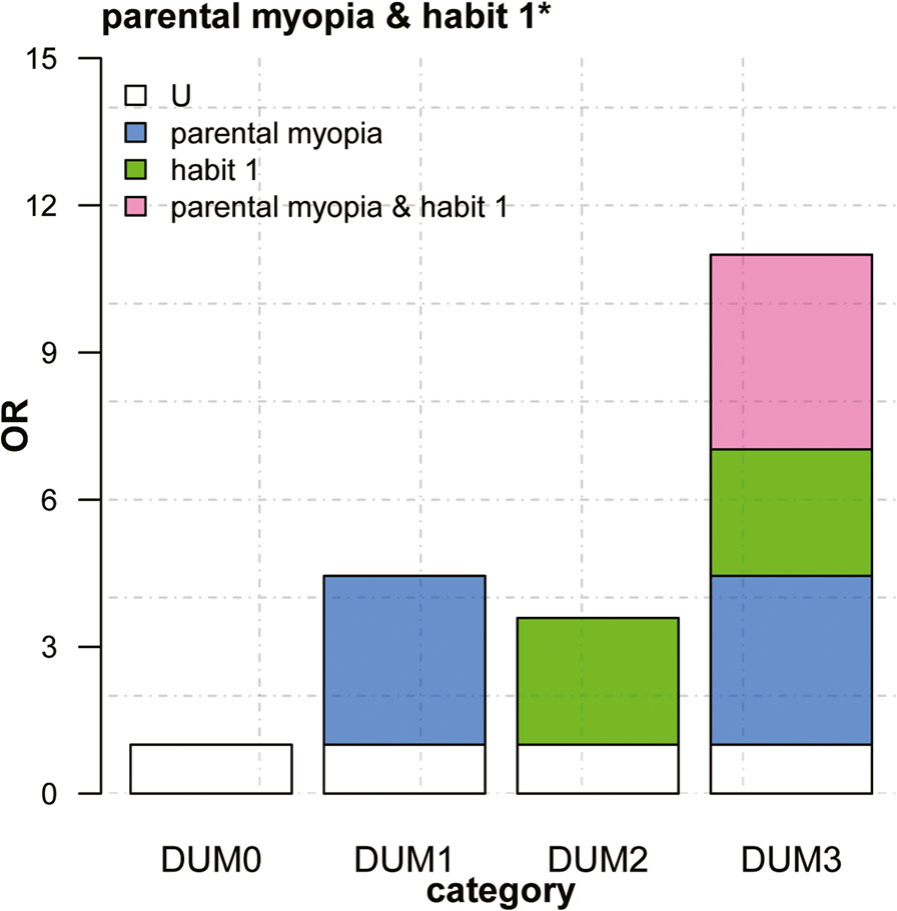
Additive interaction between parental myopia and poor eye habits when reading and writin on myopia. * poor eye habits when reading and writing

**Fig. 2:**
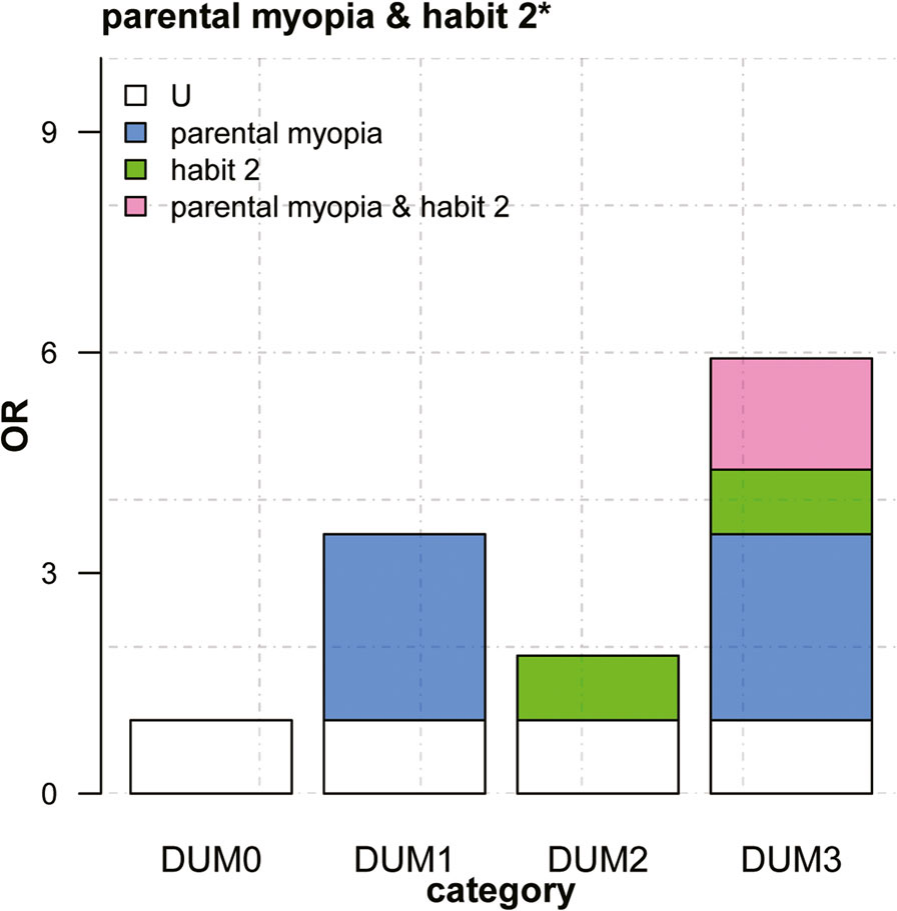
Additive interaction between parental myopia and poor reading posture on myopia. * poor reading posture

Multivariate logistic regression models were constructed to evaluate factors associated with myopia after adjusting for confounders. In multivariate models, the presence of myopia was associated with parental myopia, habit 1, and habit 2. Both models 1 and 2 were consistent (Table 3). Parental myopia and habit 1 and habit 2 were converted into three dummy variables (*DUM*). These were then used in the multivariate logistic regression models to obtain three regression coefficients in terms of dummy variables, *OR*, and covariance (Table 4). Three indicators for evaluating interaction were determined through regression coefficients and covariance: relative excess risk due to interaction (*RERI*), attributable proportion (*AP*), and synergy index (*S*) (Table 5). The judgment of additive interaction is based on *RERI*, *AP*, *S* and their 95 % confidence intervals (95 % *CI*); when the 95 % *CI* of *RERI* and *AP* does not contain 0 and the 95 % *CI* of *S* does not contain 1, it indicates that an additive interaction exists and is significant. For parental myopia and habit 1 and habit 2 in both models 1 and 2, the 95 % *CI* of *RERI* and *AP* did not contain 0, the 95 % *CI* of *S* did not contain 1, proving that there were additive interactions between them. It indicated that when parental myopia present with habit 1 and habit 2, the risk of having myopia was greater than that due to the existence of the two independently. The additive interaction diagrams of Model 2 were shown in Figs. 1 and 2.

**Table 3:** Logistic regression analysis of the correlation of parental myopia and poor reading and writing habits with students’ myopia

	*β*	*OR* (95 % *CI*)	*P*
Model 1			
Parental myopia	1.26	3.51 (3.11–3.96)	< 0.01
Habit 1	1.03	2.81 (2.28–3.46)	< 0.01
Habit 2	0.37	1.45 (1.25–1.68)	< 0.01
Model 2			
Parents myopia	1.15	3.15 (2.78–3.57)	< 0.01
Habit 1	1.01	2.74 (2.21–3.41)	< 0.01
Habit 2	0.41	1.51 (1.29–1.76)	< 0.01

Model 1: adjusted for age and sex; Model 2: adjusted for age, sex, ethnicity, and learning level; Habit 1: Poor eye habits when reading and writing; Habit 2: Poor reading posture

**Table 4:** Analysis of the additive interaction of parental inheritance and poor reading and writing habits on students’ myopia

	*β*_1_	*OR*_1_ (95 % *CI*)	*P*_1_	*β*_2_	*OR*_2_ (95 % *CI)*	*P*_2_
Parental myopia and Habit 1						
DUM 1	1.69	5.43(3.60–8.20)	< 0.01	1.49	4.44 (2.90–6.78)	< 0.01
DUM 2	1.33	3.76(2.91–4.94)	< 0.01	1.27	3.58 (2.74–4.73)	< 0.01
DUM 3	2.55	12.83(9.81–17.01)	< 0.01	2.40	10.99 (8.33–14.68)	< 0.01
Parental myopia and Habit 2						
DUM 1	1.47	4.38 (3.38–5.68)	< 0.01	1.26	3.53 (2.70–4.62)	< 0.01
DUM 2	0.65	1.91 (1.60–2.30)	< 0.01	0.63	1.88 (1.56–2.27)	< 0.01
DUM 3	1.89	6.59 (5.43–8.04)	< 0.01	1.78	5.92 (4.84–7.27)	< 0.01

Model 1: adjusted for age and sex; Model 2: adjusted for age, sex, ethnicity, and learning level; Habit 1: Poor eye habits when reading and writing; Habit 2: Poor reading posture

**Table 5:** Evaluation index for additive interaction between parental inheritance and poor reading and writing habits

	*RERI* (95 % *CI*)	*AP* (95 % *CI*)	*S* (95 % *CI*)
Parental myopia and Habit 1			
Model 1	254.23 (36.39–427.06)	0.97 (0.95–0.98)	36.32 (22.86–57.71)
Model 2	167.26 (19.37–315.15)	0.96 (0.94–0.98)	28.83 (18.13–45.83)
Parental myopia and Habit 2			
Model 1	49.94 (20.93–78.95)	0.90 (0.87–0.94)	12.63 (9.48–16.83)
Model 2	34.87 (13.83–55.92)	0.89 (0.85–0.92)	11.23 (8.49–14.86)

Model 1: adjusted for age and sex; Model 2: adjusted for age, sex, ethnicity, and learning level; Habit 1: Poor eye habits when reading and writing; Habit 2: Poor reading posture

Parental myopia and habit 1 and habit 2 were entered into the logistic regression model together to obtain the regression coefficient, *OR*, and *P*-value for three variables. After adjusting the relevant variables, if the *P*-value for parental myopia and poor reading and writing habits was less than 0.05, then it can be considered that parental myopia and poor reading and writing habits have a multiplicative interaction on myopia. However, the *P* for parental myopia and habit 1 was less than 0.05 only in Model 1, while in Model 2, the *P* was greater than 0.05, and the *P* for parental myopia and habit 2 was greater than 0.05 in models 1 and 2; therefore, there were no multiplicative interactions between parental myopia and habit 2 (Table 6).

**Table 6:** Analysis of multiplicative interaction between parental myopia and poor reading and writing habits on students’ myopia

	*β*_1_	*OR*_1_ (95 % *CI*)	*P*_1_	*β*_2_	*OR*_2_ (95 % C*I*)	*P*_2_
Parental myopia and Habit 1						
Parental myopia	1.69	5.43 (3.60–8.20)	< 0.01	1.49	4.44 (2.90–6.78)	< 0.01
Habit 1	1.33	3.76 (2.91–4.94)	< 0.01	1.27	3.58 (2.74–4.73)	< 0.01
Parental myopia and Habit 1	-0.47	0.63 (0.41–0.96)	0.05	–0.37	0.69 (0.44–1.08)	0.10
Parental myopia and poor reading and writing habits (2)						
Parental myopia	1.47	4.38 (3.38–5.68)	< 0.01	1.26	3.53 (2.70–4.62)	< 0.01
Habit 2	0.69	1.91 (1.60–2.30)	< 0.01	0.63	1.88 (1.56–2.27)	< 0.01
Parental myopia and Habit 2	-0.23	0.79 (0.59–1.05)	0.11	–0.11	0.89 (0.66–1.21)	0.46

Model 1: adjusted for age and sex; Model 2: adjusted for age, sex, ethnicity, and learning level; Habit 1: Poor eye habits when reading and writing; Habit 2: Poor reading posture

## Discussion

This school-based eye survey documented that approximately 19.9 % of primary school students, 44.5 % of junior high school students, and 72.0 % of senior high school had myopia. Although this was lower than the prevalence in most other provinces and cities in China [4], there was a clear upward trend compared with the findings from our research group’s survey in 2010 [15, 17]. This upward trend can also be seen in other studies in China [19]. According to Dong et al. [20], the prevalence of myopia in 2050 among children and adolescents aged 3–19 years is estimated to reach 84 %. There is no doubt that if no measures are taken, the rates of myopia in Urumqi primary and middle school students will continue to rise.

Many studies have concluded that girls have a higher prevalence of myopia than boys [20–23], consistent with our finding. The reason is that girls’ learning attitudes are more active than those of boys, and they have less outdoor activities. Sherwin et al. [24] found that increasing outdoor activity time might be a strategy to reduce myopia in children and adolescents. Outdoor activities can increase exposure to the sun, and light can stimulate the synthesis and release of dopamine in the retina, which shortens the axis of the eye [25, 26], thus preventing myopia. Our survey also found that the prevalence of myopia among Han students was higher than that among other ethnic groups. Previous studies by Tang et al. [27] reached the same conclusion. The reason for this difference may be owing to differences in lifestyle between the Han and ethnic minority populations in Urumqi. For example, grasp is a staple food unique to Xinjiang. It is made of rice and carrots; carrots are rich in vitamin A, which can prevent corneal dryness and degeneration, eliminate eye fatigue, and protect visual function [28].

In addition, genetic factors may also be responsible for the difference in the prevalence of myopia between sexes and ethnicities, the higher the heritability, the more important the role of genetic factors in the pathogenesis of a disease [15, 29]. In our study, the heritability in the Han population was higher than that in the other ethnic groups, and in girls was higher than that in boys, these indicating that myopia has a high genetic susceptibility in Han and girl population. At present, many studies have shown that having myopic parents increases the risk of myopia in children. For example, a study by O’Donoghue et al. [30] on children aged 12–13 years in Northern Ireland showed that children who have one or both parents with myopia were 2.91 and 7.79 times more likely, respectively, to develop myopia than children whose parents were not myopic. Moreover, some studies have shown that the severity of myopia in parents and the number of myopic parents are factors that lead to aggravation of myopia in children [31, 32]. The multivariate logistic regression in this study also found that parental myopia increases the risk of myopia in children. At present, 25 gene loci are clearly related to the pathogenesis of myopia (MYP1-25) [33–35]. With the continuous development of molecular genetics and related detection technologies, an increasing number of genes related to myopia will be found; however, there is still a lack of genetic surveys on myopia in Xinjiang.

In China, students are pressurized to study; therefore, they spend more time reading and writing. Huang et al. [36] search indicated that the time spent reading and writing with the object in close proximity to the eyes was associated with the risk of myopia. If students have poor reading and writing habits, the risk of myopia may be further increased. In this study, multivariate logistic regression found that habit 1 and habit 2 will increase the risk of myopia. In addition to multivariate logistic regression analysis, we also analyzed the potential additive and multiplicative interaction between parents’ heredity and habit 1 and habit 2. The definition of interaction is that when two or more risk factors exist at the same time, the risk of the disease is different from the sum of the individual effects of each risk factor [37]. This study found that there was an additive interaction between parental myopia and the habit 1 and habit 2; some scholars have proposed that it is more scientific to use the additive model to analyze biological interaction [38, 39].

In our analysis results, habit 1 resulted in a higher risk of myopia in students than habit 2, and the additive interaction between parental myopia and habit 1 was also higher than habit 2. In the habit 1 group, because of the close proximity of reading and writing, the closer the eyes are to the objects, the greater the power used by the ciliary muscles, and the longer the working time in close proximity. Pupils with a reading / writing distance of less than 30 cm are often in a state of ciliary contraction, thus predisposing to the development of myopia. Whereas masking the eyes on one side when writing, or tilting the body when reading and / or writing, predisposes to inconsistencies in the distance between eyes and books. It may also be responsible for some of the pupils not having the same visual acuity in both eyes. On the other hand, incorrect poses, on the other hand, lead to inconsistent distances between the eyes and the book. We speculate that lying down, reading and writing in a walking or moving state, attention is distracted more and requires higher intensity eye use, predisposing to asthenopia. This may be the reason why habit 2 lead to the rising risk of myopia in students.

Combined with this study, our conclusions suggest that parental myopia makes children more susceptible to poor reading and writing habits, leading to a greater risk of myopia. It also reminds us that when students enter school, we should investigate whether their parents have myopia, and pay attention to students whose parents have myopia to actively correct poor reading and writing habits to reduce the risk of myopia in students.

There are still some limitations to the present study. First, because of the large number and considering the feasibility of the investigation, tropicamide with a faster onset and shorter recovery time was selected for cycloplegia and mydriasis optometry during eye examination. This has the potential to lead to an overestimation of the diagnostic rate of myopia. In addition, there is no comment on high myopia, glass use, or hours of study and outdoor activity. This may lead to a dearth of inquiry into risk factors for myopia. In this study, an interaction analysis was conducted to explore the contribution of a genetic history of myopia to myopia among students induced by poor reading and writing habits. This suggests that a focus should be placed on students with a family history of myopia.

## Conclusions

At present, the overall myopia rate of students in Urumqi is relatively high, and myopia has a high genetic susceptibility. For students with parents who have myopia, we should focus on interventions. For students with poor reading and writing habits, schools and parents should remind students to correct these habits to reduce the risk of developing myopia.

## Supplementary Information

**Additional file 1.**

## Data Availability

The datasets used and/or analyzed during the current study available from the corresponding author on reasonable request.

## Abbreviations

CI: Confidence interval
OR: Odds ratio
DUM: Dummy variables
RERI: Relative excess risk due to interaction
AP: Attributable proportion
S: Synergy index
