# Supplementary material for: Influence of the interaction between parental myopia and poor eye habits when reading and writing and poor reading posture on prevalence of myopia in school students in Urumqi, China
Source: BMC Ophthalmol. 2021 Aug 14;21:299. doi: 10.1186/s12886-021-02058-3 (PMC8364037; doi:10.1186/s12886-021-02058-3)
Supplement: Supplementary file 1 — Additional file 1. [file 12886_2021_2058_MOESM1_ESM.docx]

No.: W


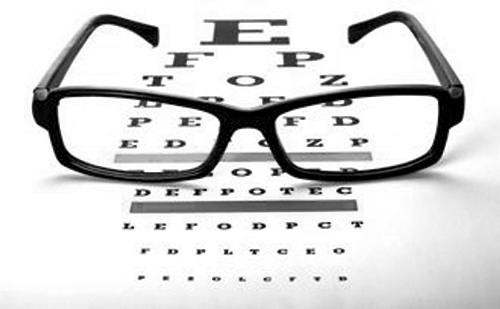


# The Influence of Adolescent Myopia in Urumqi

Factor questionnaire

Urumqi juvenile myopia Factors Questionnaire

Urumqi School of Public Health, Xinjiang Medical University

**Parents' informed consent form of questionnaire on influencing factors of juvenile myopia**

**Parents: Hello!**

The investigation shows that the myopia of children and adolescents in our country is in the trend of early age and continuously rising. The evidence-based ophthalmology study shows that there is no cure for: Myopia at present, which is the most prominent problem endangering the health of children and adolescents in our country. Protecting your child's health is not only your responsibility, but also the responsibility of our researchers. For this reason, we set up a juvenile myopia prevention and treatment team, professional researchers will have a targeted in-depth analysis of eye environment, academic burden, life habits of children's visual threat, to ensure the healthy growth of children.

1. Purpose of the study: We will analyze the potential factors threatening children's visual health through the data you fill in, and find out the ways and means to reduce the possibility of children's illness. The study was approved by the Ethics Committee of the First Clinical Medical College of Xinjiang Medical University.

II. Examination (research) content: (1) Questionnaire: Including general condition, vision condition, eye use condition, diet habit, etc. (2) Optometry examination: If your child's myopia is relatively serious or early onset or has family history, further measurement of ophthalmology may include optometry or mydriasis optometry, fundus optometry, and OCT. ③3 ml screening of micronutrients associated with myopia in venous blood (optional).

3. Risk: During your physical measurement and questionnaire survey, the eye examination is non-contact and there is no risk. It only takes part of your time. If blood sampling is also very safe, please take part in the survey.

IV. Benefits:

All patients involved in the study will be free of charge for testing. We will feed back the survey results to you in a timely manner to find ways and means to reduce the likelihood of the child's illness.

V. Confidentiality:

Respect the privacy of the child and his or her family, except for authorized researchers, without their consent. At no time shall the patient's history, examination information or findings be disclosed to others. The test results and data collected are for scientific research only.

If you have any questions about the design, process and results of this study, please contact us at 18690152215 13565921910 and we will try to answer your questions. Thank you and your family for your trust and support! If you are a minority, you will be answered by a doctor familiar with your language.

I am aware of the purpose, content, significance and risks of this study and have volunteered to participate in this study. Signature of guardian, contact number:

Dear classmates,

Hello! In order to understand the eyesight of the primary and middle school students in Urumqi and formulate effective prevention and control strategy, we investigated the subjects of prevention and treatment of juvenile myopia. We would like to thank you very much for your participation in this survey as a student representative of the school. Please kindly provide your views and comments and hope to receive your support. Students in the process of filling out the questionnaire, fill in the blanks to fill in the information above the horizontal line, select the question in front of the selection of the tick. We are honored to listen to your thoughts. Thank you!

**I. Basic information**

| 1.1. You are currently enrolled in the school grade class, and your name is. |
| --- |
| 1.2 Your sex: □ male/female 1.2a.Ask your mother or family how you were born: Cesarean section |
| 1.3 Your Nationality: □ Han, Uygur, Kazak, Hui, Mongolian, Other |
| 1.4 Your Birth Weight: (G) Your Birth Height: _ _ (cm) Your Last Weight: (Kg) Test Time: () Years  () months; You last measured a height of: (Cm); Test time: () years () months |
| 1.5 The annual average pre-tax income of parents is about: 50000-100000-100000-200000-200000-400000 or above |
| 1.6 .. Your grades in the class are: □ excellent? Good? Middle and top? Middle? Middle and bottom? Poor |
| 1.7 Please select the interesting class you have attended (multiple choices are allowed): Piano? Other musical instruments? Painting calligraphy? Sports? Other words? Not taken  Plus any interest classes. |
| - 1. .Your date of birth is, and your age is, you or your parents can contact at:   2. Your current permanent address is District (County) Road (Village, Street, Lane) Community (Name) |
| 1.10 Does your mother often live at the above address during her pregnancy? Yes? No |
| 1.11 The date of investigation shall be filled in by: I, my parents, other guardians |

**II. Child feeding and family members**

| 2.1 . Are you the only child?: □ Yes? No | | |
| --- | --- | --- |
| - 1. A. If "no," you have a brother and sister, a brother and sister.   2. **Among family members, those who smoke are: No, father, mother, grandfather, grandmother, other personnel**   3. **The smoking of the family members who live together during the pregnancy of the mother is: * No * Father * Mother * Grandfather/Grandfather * Grandmother/Grandmother * Other persons**   4. **Among family members, those who drink alcohol are: No, father, mother, grandfather/grandfather, grandmother/grandmother, other personnel** | | |
| 2.5 . Ask mother about duration of exclusive breastfeeding: | | |
| **? No breastfeeding? Less than one month □≥1 months and <4 months □≥4 months and <□≥6 months and <1 years □≥1 years or more** | | |
| 2.6 . The time you start a nursery or kindergarten: | | |
| - 1. The number of classes in your school is approximately: □30 or less □30-50 or more □50 or more   2. Your mother's degree: Primary school or below, secondary school or below, college or undergraduate, master's degree or above | | |
| 2.9 . Your father's degree: | Primary school and below? Secondary school or below? Junior college or undergraduate? Master's degree or above |  |

**III. Vision and related conditions**

**3.1a left uncorrected visual acuity: Or degrees:**

- 1. B Right uncorrected visual acuity: Or degrees:
  2. **Do you have myopia? Yes No If you have myopia, skip directly to question 3.4**
  3. **If there is no myopia, your vision is normal, hyperopia, weak or strabismus, other(Go to question 3.9)**
  4. **If myopia, do you have other eye diseases (such as trachoma, strabismus, etc.) besides myopia 🖾 Yes? No**
  5. The change of your glasses is within: □1 years □1-2 years and more than □2 years

**3.6 Did you receive relevant intervention treatment after myopia? Yes No If yes, please indicate which treatment**

- 1. The age at which your eyesight begins to deteriorate is, the age at which you wear glasses.
  2. **You think the reason for your near-sightedness may be that you can choose more than one: Parents' transmission, too much learning burden, too much light, too much light or too much sunlight, too long time playing with electronic products, malnutrition, eye diseases, reading while riding in a car, picky food, partial food, bad rest or sleep, incorrect position of holding a pen, not knowing what else to write**
  3. **Is your family near-sighted? Left eye degree of father myopia, right eye degree; Left eye degree of mother's myopia, right eye degree; Brother:**

**Myopia, not myopia; Sister: Myopia, not myopia; Grandparents: Myopia, not myopia; Grandparents: Myopia, not myopia; Uncle (father's biological brother): Myopia, not myopia; Aunt (father's real sister): Myopia, not myopia; Aunt (mother's real sister): Myopia, not myopia; Uncle (mother's real brother): Myopia, not myopia; Where 🖾 above 600 degrees None,**

**IV. Eye behavior habits:**

| **4.1 When you are reading, your head is too low and your eyes are less than 30 cm: □ away from the book.** |
| --- |
| **There is a habit of reading a book, cell phone, or reading, often and occasionally** |
| **4.3 The position of the pen is too low or the finger is blocking the line of sight.** |
| **4.4. There is body or head skew when writing. This is often done. Occasionally it is hardly done.** |
| **4.5 In the case of dark light, turn on the light during reading and writing. It is often done. Occasionally, it is hardly done.** |
| **4.6. There is a habit of reading while walking or riding. It is often done. Occasionally it is hardly done.** |
| **4.7 After 1 hour of continuous reading and writing, rest the eyes:** |
| **4.8 Your usual learning lighting is? Desk lamp? Roof light source? Both are on** |
| **4.9 Do you do eye exercises every day at school?Do it every day sometimes, sometimes not** |
| **4.10 You do eye exercises: □l times, □2 times, □3 times or more than three times a day.** |
| **4.11 When you read and write, the average duration of each time is about: □ less than 1h □1h~2h □2h-3h □ more than 3h.** |
| **4.12 Do you feel tired eyes? Yes, often. Occasionally. Hardly ever.** |
| **Do you like to look beyond? Yes, often. Occasionally. Hardly ever.** |

**V. Academic problems:**

| **5.1 What do you usually do during breaks?Activity outside the classroom, homework inside the classroom, sleeping inside the classroom, chatting inside the classroom** |
| --- |

| **Your best course is: Chinese, Mathematics, English, Physical Education, Other (please write)** |
| --- |
| **5.3 Can you see the words on the blackboard of the classroom when the light is bad at night or on cloudy days?Energy** |
| **5.4 How much time do you need to do your homework and preview your lessons every day?Less than 1h □1h~2h □2h-3h: □ > 3h** |
| **5.5 Participating in weekly tutorial or extracurricular study: None, hours.** |
| **5.6 Can physical education in the school be on time? Almost on time, occasionally, often** |
| **5.7 At weekends, the most common thing you do is to do: □ homework, make-up lessons, take part in fun classes, go out to play with your parents or classmates, go shopping, take part in sports, watch TV, go online or play tablet games, go to relatives, take part in various community activities, and others** |

**VI. Other Factors:**

| **6.1. Breakfast in the semester? Every day? Occasionally? Frequently?** | | | | |
| --- | --- | --- | --- | --- |
| - 1. **Partial eating habit:? No? Yes, if any, please select? Meat food? Vegetarian food? Sweet food**   2. How often do you drink milk per week? Hardly? 1-2 times per week? 2-3 times per week? 4-5 or more times per week   3. How often do you drink yogurt per week? Almost no? 1-2 times per week? 2-3 times per week? 4-5 or more times per week   4. What you prefer is: Hot food, sweet food, salty food, snack food, dried fruit 🖾 drink   5. The number of times you eat food in a week is probably: Almost no food in a week is less than 2 times in a week is more than 2 times in a week | | | | |
| **6.7 Among the following main foods, your favorite foods are: □ noodles, noodles, steamed bread, bread, wonton and other noodles? Rice and other noodles? Corn and its products? Others:** | | | | |
| **Of the following non-staple foods, your favorite is:**  **Carrots, meat, milk, poultry, seafood, fresh vegetables, fruits, pickled food, sweets, other** | | | | |
|  | **6.9. How many hours do you sleep?** | During term  School days | **Less than 6h □6-8h □8~10 h □ > 10 h** |  |
|  |  | During term  Weekend | **Less than 6h □6-8h □8~10 h □ > 10 h** |  |
|  |  | Winter and summer holidays | **Less than 6h □6-8h □8~10 h □ > 10 h** |  |
|  | **6.10. Your daily television time?** | During term  School days | **Not seen less than 1h □1h~2h □2h-3h:** |  |
|  |  | Mid-term weekend | **Not seen less than 1h □1h~2h □2h-3h:** |  |
|  |  | Winter and summer holidays | **Not seen less than 1h □1h~2h □2h-3h:** |  |
|  | **6.11. Your daily phone/tablet time** | During term  School days | **Less than 1h □1h~2h □2h-3h □ > 3h** |  |
|  |  | Mid-term weekend | **Less than 1h □1h~2h □2h-3h □ > 3h** |  |
|  |  | Winter and summer holidays | **Less than 1h □1h~2h □2h-3h □ > 3h** |  |
|  | **6.12. Your daily computer time?** | During term | **Less than 1h □1h~2h □2h-3h □ > 3h** |  |

|  |  | School days |  |  |
| --- | --- | --- | --- | --- |
|  |  | During term  Weekend | **Less than 1h □1h~2h □🖾2h-3h □ > 3h** |  |
|  |  | Winter and summer holidays | **Less than 1h □1h~2h □2h-3h □ > 3h** |  |
|  | **6.13. Your daily outdoor time?** | During term  School days | **Less than 30 min □0.5~1h □1h-2h □ more than 2h** |  |
|  |  | Mid-term weekend | **Less than 30 min □.5~1h □1h-2h □ > 2h** |  |
|  |  | Winter and summer holidays | **Less than 30 min □0.5~1h □1h-2h □ more than 2h** |  |
| - 1. **Excluding physical education classes and extracurricular activities required by the school, the number of times you take part in physical education activities per week (that is, lasting for more than 30minutes each time)? Not taking part in □1 times, □2 times, □3 times, □4 times or more**   2. **You feel the school desks and chairs: Very fit. Too high. Too short.** | | | | |
| **6.16. You feel the color of the pictures and words in the textbook:** | | | | |
| **6.17. Parents advise you to look at the distance or close your eyes and relax.** | | | | |
| **6.18. The way you wash your face is to wash your face with flowing water** | | | | |
| **6.19. Do you take part in table tennis? Yes, often? Occasionally? Hardly** | | | | |
| **6.20. Do you participate in other ball sports? Yes? No. If yes, please indicate which type** | | | | |
| - 1. **The way you go to school is as follows: () Parents accompany you by transportation () You accompany yourself by transportation () You walk () Parents or classmates accompany you walk ()**   2. Do you turn on the light while you sleep? Not open often open occasionally open all the way | | | | |

**VII. Investigation of Natural Light in Classroom**

| **.Please describe whether you can see the blackboard clearly.** | | |
| --- | --- | --- |
| **7.1 Please evaluate whether the natural light can clearly see the words on the blackboard when the classroom lights are off.**  **It's enough. It's almost enough. It's not enough. It's not enough.** | | |
| **7.2 Please assess whether the room light is clear enough to see the words on the blackboard when the classroom lights are on.**  **It's enough. It's almost enough. It's not enough. It's not enough.** | | |
| **7.3 When the light is not turned on, which positions of the blackboard can you see clearly?**  **Near window side, blackboard middle side, near door side, not easy to see** | | |
| **7.4 When turning on the light, which positions of the blackboard can you see clearly?**  **Near window side, blackboard middle side, near door side, not easy to see** | | |
| **7.5 Please indicate where you were in the classroom when you completed the questionnaire:**  **Window Side Front Side Rear Side Door Side Front Side Door Side Rear Side** | **Anterior middle part** | **Middle rear** |
| **7.6. Do you think the blackboard is reflective when the light is not turned on?**  **Severe reflective, very reflective, general, small reflective** | **Completely non-reflective** |  |
| **7.7 Do you think the blackboard is reflective when the lights are turned on?** | | |

| **Severe reflective, very reflective, general, small reflective, not reflective at all** |
| --- |
| **7.8 In general, does this reflection prevent you from reading the words on the blackboard?**  **? Serious impact? Very impact? Minor impact? No impact at all** |
| **7.9 Is this the natural light or the light?**  **Complete natural light? Natural light one more point? Half half? Electric light one more point? Complete electric light** |
| **7.10 Are you satisfied with the light on the classroom blackboard?**  **Very Satisfied Satisfaction General Dissatisfaction Very Dissatisfied** |

**VIII. Lighting situation of classroom:**

| **.Please describe the lighting in your classroom** | |
| --- | --- |
| **8.1 Do you think the windows in the classroom are big enough?**  **Very sufficient** | **Not enough** |
| **8.2 Does the light outside the window make you uncomfortable?**  **Very Comfortable Comfortable General Uncomfortable** | **Very uncomfortable** |
| **8.3 When the light is not turned on normally, do you think the indoor light is enough to read and write on the seat?**  **It's very enough. It's enough. It's not enough. It's not enough.** | |
| **8.4 When turning on the light, will the reflection of electric light often hinder and affect your reading and writing?**  **? Serious impact? Very impact? Minor impact? No impact at all** | |
| **8.5 When the light is not turned on, are you satisfied with the light on your desk?**  **Very Satisfied Satisfied General Dissatisfied** | **Very dissatisfied** |
| **8.6 When turning on the light, are you satisfied with the light on your desk?**  **Very Satisfied Satisfied General Dissatisfied** | **Very dissatisfied** |
| **8.7 Except when the teacher uses video equipment (TV, projector, etc.), do you often use curtains?**  **Very often. Very often. Very little. Not at all.** | |
| **8.8 In the classroom, do you want to read and write with more natural light or more electric light?**  **Complete natural light? Natural light one more point? Half half? Electric light one more point? Complete electric light** | |
| **8.9 Are you satisfied with the light in the classroom (natural light with the light)?**  **Very Satisfied Satisfaction General Dissatisfaction Very Dissatisfied** | |
| **8.10 If you have any opinions and suggestions on the quality of lighting in the classroom, please state the following. (Your opinion will be strictly confidential)** | |

**Thank you again for your help and support in our work! Contact person: Shi Haonan 18690152215**

**Prevention and treatment of juvenile myopia**
